# Comparative proteomics of *Geobacter sulfurreducens* PCA^T^ in response to acetate, formate and/or hydrogen as electron donor

**DOI:** 10.1111/1462-2920.15311

**Published:** 2020-11-20

**Authors:** Monir Mollaei, Peer H. A. Timmers, Maria Suarez‐Diez, Sjef Boeren, Antonie H. van Gelder, Alfons J. M. Stams, Caroline M. Plugge

**Affiliations:** ^1^ Wetsus, European Centre of Excellence for Sustainable Water Technology Leeuwarden The Netherlands; ^2^ Laboratory of Microbiology Wageningen University & Research Wageningen The Netherlands; ^3^ Laboratory of Systems and Synthetic Biology Wageningen University & Research Wageningen The Netherlands; ^4^ Laboratory of Biochemistry Wageningen University & Research Wageningen The Netherlands; ^5^ Centre of Biological Engineering University of Minho Braga Portugal

## Abstract

*Geobacter sulfurreducens* is a model bacterium to study the degradation of organic compounds coupled to the reduction of Fe(III). The response of *G. sulfurreducens* to the electron donors acetate, formate, hydrogen and a mixture of all three with Fe(III) citrate as electron acceptor was studied using comparative physiological and proteomic approaches. Variations in the supplied electron donors resulted in differential abundance of proteins involved in the citric acid cycle (CAC), gluconeogenesis, electron transport, and hydrogenases and formate dehydrogenase. Our results provided new insights into the electron donor metabolism of *G. sulfurreducens*. Remarkably, formate was the preferred electron donor compared to acetate, hydrogen, or acetate plus hydrogen. When hydrogen was the electron donor, formate was formed, which was associated with a high abundance of formate dehydrogenase. Notably, abundant proteins of two CO_2_ fixation pathways (acetyl‐CoA pathway and the reversed oxidative CAC) corroborated chemolithoautotrophic growth of *G. sulfurreducens* with formate or hydrogen and CO_2_, and provided novel insight into chemolithoautotrophic growth of *G. sulfurreducens*.

## Introduction

*Geobacter* species play an important role in organic matter degradation coupled to Fe(III) reduction, and are well‐known for making electrical contacts with external electron acceptors, and also with other microorganisms (Lovley *et al*., 1987; Lovley, 2011, 2017). *Geobacter* species are applied in bioelectrochemical systems owing to their capacity to transfer electrons to and from electrodes, and their remarkable respiratory versatility (Bond *et al*., 2002; Holmes *et al*., 2004). Moreover, some *Geobacter* species can degrade organic pollutants such as benzene and phenol, and transform Co(III), Fe(III), Mn(IV), U(VI) and V(V) oxides of which some are toxic or radioactive (North *et al*., 2004; Ortiz‐Bernad *et al*., 2004; Schleinitz *et al*., 2009; Zhang *et al*., 2014).

*Geobacter sulfurreducens* PCA has served as a model microorganism in different studies where it is often grown with acetate as the electron donor and carbon source and Fe(III) citrate as the electron acceptor (Segura *et al*., 2008; Yang *et al*., 2010; Speers and Reguera, 2012). *G. sulfurreducens* degrades acetate via the citric acid cycle (CAC), whereas its carbon assimilation starts from acetyl‐CoA via pyruvate through gluconeogenesis (Galushko and Schink, 2000; Yang *et al*., 2010). In contrast, less consistent information is available about the growth of *G. sulfurreducens* on formate and/or hydrogen. Although *G. sulfurreducens* is known to reduce Fe(III) with formate or hydrogen as electron donor, addition of low amounts of acetate was necessary for carbon assimilation (Coppi *et al*., 2004; Speers and Reguera, 2012). Former genomic analysis of *G. sulfurreducens* has predicted formate catabolism by a periplasmic membrane‐bound formate dehydrogenase (Fdh) (Methe *et al*., 2003). Formate assimilation was predicted to be mediated by a pyruvate formate lyase (Pfl), which needs acetyl‐CoA and formate to produce pyruvate and CoA (Segura *et al*., 2008). The acetyl‐CoA needed for the assimilation derived from the acetate (0.1 mM) supplemented to the cultures (Speers and Reguera, 2012). Hydrogen oxidation by *G. sulfurreducens* is catalysed by Hyb, a periplasmically oriented respiratory uptake hydrogenase (Coppi *et al*., 2004). When traces of acetate (~1 mM) are available as carbon source, the energy conservation from hydrogen oxidation decreases the demand for acetyl‐CoA oxidation through the CAC, and more acetate is channelled towards pyruvate synthesis for anabolic reactions (Yang *et al*., 2010). A recent study revealed chemolithoautotrophic growth of *G. sulfurreducens* on formate or hydrogen and CO_2_, underpinning the role of the reversed oxidative CAC in CO_2_ reduction using a bidirectional citrate synthase (Zhang *et al*., 2020). Citrate synthase of *G. sulfurreducens* was previously proposed to function only in oxidative CAC (Galushko and Schink, 2000). However, the carbon metabolism of *G. sulfurreducens* when grown with hydrogen and/or formate with CO_2_ or acetate as carbon source has not been fully understood.

The aim of this study was to gain insight in metabolism of *G. sulfurreducens* in response to a variety of electron donors. We performed physiological studies using either acetate, formate, hydrogen or different combinations of these electron donors with Fe(III) citrate as electron acceptor. Acetate was never supplied as carbon source to the cultures growing with formate or hydrogen and Fe(III) citrate was always used as electron acceptor. Comparative proteomic analysis was used to understand the response of *G. sulfurreducens* to individual electron donors or a mixture of all three.

## Results and discussion

### Growth phenotypes using different electron donors

**Fig 1:**
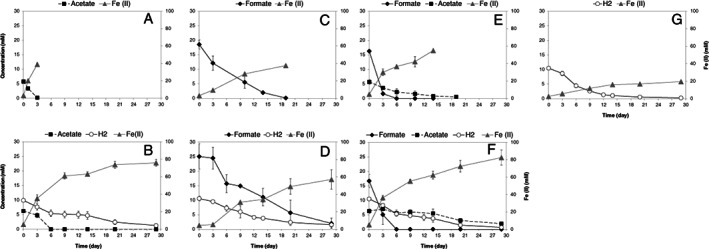
Growth of *G. sulfurreducens* with different electron donors and Fe(III) citrate as electron acceptor. A Acetate. B. Acetate + hydrogen. C. Formate. D. Formate + hydrogen. E. Acetate + formate. F. Acetate + formate + hydrogen. G. Hydrogen. The concentration of hydrogen was shown as mmol/L liquid. Data are means of triplicates.

*G. sulfurreducens* oxidized acetate, formate, hydrogen, and different combinations of these substrates coupled to the reduction of Fe(III) to Fe(II) (Fig. 1). Acetate was depleted within 3 days (Fig. 1A), whereas formate was degraded within 19 days (Fig. 1C). Hydrogen with CO_2_ as the sole carbon source was depleted within 30 days (Fig. 1G).

When hydrogen was used in combination with acetate (Fig. 1B), formate (Fig. 1D) and acetate plus formate (Fig. 1F), all substrates were used throughout the growth period. However, in the presence of hydrogen, consumption of acetate and/or formate was slower (Fig. 1 B, D and F) than the corresponding cultures in absence of hydrogen (Fig. 1 A, C and E). This points to a negative impact of hydrogen on consumption of acetate and formate. Hydrogen is known to induce the expression of HgtR (GSU3364), a repressor which downregulates CAC enzymes such as citrate synthase (GltA), which in turn lowers acetate oxidation in the CAC (Ueki and Lovley, 2010). HgtR was not detected in our results. Surprisingly, when acetate and formate were used concurrently, formate was the preferred electron donor over acetate (Fig. 1E). As such, the electrons released from formate consumption (16.25 × 2 = 32.5) after 6 days is more than those from acetate consumption (3.46 × 8 = 27.7). This is also the case in Fig. 1F after 6 days. Formate was depleted faster in the presence of acetate (in 10 days, Fig. 1E) than when formate was the sole electron donor (in 19 day, Fig. 1C). This preferential consumption of formate over acetate by *G. sulfurreducens* has not been described before. A possible explanation could be hydrogen formation from formate, and subsequent downregulation of the CAC enzymes by hydrogen (Ueki and Lovley, 2010).

In the presence of acetate (Fig. 1 E, B and F), total Fe(II) production was more than the corresponding cultures without acetate (Fig. 1 C, G and D). Therefore, acetate was used as an electron donor throughout all the conditions. For instance, in the mix condition when acetate is present in addition to formate and hydrogen (Fig. 1, F, day 3), 31.06 ± 2.18 mol Fe(II) was produced per 27.70 ± 4.82 mol of electrons released from substrates. This higher Fe(II) production indicates that acetate might also serve as an additional electron donor in presence of hydrogen and formate.

A comparison of electron donor consumption and Fe(II) production at the end of each batch culture showed that the electron recovery was not 100% (Table 1). This indicates that a part of the electrons released was used for biomass production.

**Table 1:** Stoichiometry of the reactions for *Geobacter sulfurreducens* under different growth conditions. The results are the average of three replicate incubations.

Growth conditions	Electrons released from substrates (moles)^a^	Electrons used for Fe(III) reduction (moles)^b^	Electrons used for biomass production (moles)^c^	Electron recovery (%)^d^	Mole of Fe(II) produced/ mol of single electron donor consumed
Acetate	44.57 ± 3.12	35.77 ± 1.67	8.79 ± 2.81	80.44 ± 5.01	6.9 ± 0.6
Formate	36.91 ± 3.26	34.44 ± 1.15	2.47 ± 3.06	93.70 ± 7.45	1.66 ± 0.53
Acetate + formate	73.69 ± 6.55	50.13 ± 0.71	23.56 ± 6.90	68.39 ± 6.26	–
Hydrogen	20.41 ± 0.42	17.51 ± 0.67	2.89 ± 1.06	85.87 ± 4.85	1.75 ± 0.21
Hydrogen + formate	64.20 ± 4.69	52.69 ± 2.93	12.57 ± 1.98	80.80 ± 1.58	–
Hydrogen + acetate	70.38 ± 3.59	67.09 ± 4.23	3.29 ± 1.32	95.68 ± 10.56	–
Hydrogen + acetate + formate	87.61 ± 9.54	77.31 ± 7.82	10.30 ± 4.73	88.39 ± 5.09	–

Mean ± standard deviation for three cultures.

^**a**^ Calculated based on the substrate(s) converted at the end of batch cultures multiplied by the number of electrons to be released from each substrate's complete oxidation as presented in Thauer *et al*. (1977).

^**b**^ Calculated based on the amount of Fe(II) produced at the end of the batch cultures.

^**c**^ Excess of electrons from substrate(s) oxidation after Fe(III) reduction.

^**d**^ Amount of electrons used to produce Fe(II) compared with the amount of electrons derived from substrate(s).

### Proteomic analyses

The genome of *G. sulfurreducens* PCA is 3.8 Mb in size with 3432 open reading frames and contains 3430 protein coding genes (Butler *et al*., 2012). From the four growth conditions studied, a total of 1584 proteins were detected (46.2% of the total proteins) with two or more peptides of which at least one is unique (Supplementary file 1). Among these, 1204 proteins (76% of the detected proteins) were shared in all the studied conditions (Supporting Information Fig. S1). The total proteins identified in any of the individual four growth conditions ranged from 1297 (growth with formate) to 1526 (growth with acetate). Principal component analysis (PCA) revealed that the protein abundance patterns were reproducible among the triplicates of a given growth condition (Supporting Information Fig. S2A, B) except for one replicate of the mix condition (Mix_2) which contained all three electron donors. This was likely due to a difference in sample preparation e.g. that the cells were not lysed completely. Therefore, this replicate was not considered for further analysis (heatmap, PCA and Venn diagram).

### The central metabolic network of *G. sulfurreducens* in response to the electron donor changes

Acetate has been the main substrate used in studies of the metabolism of *G. sulfurreducens*. In contrast, little attention has been given to formate and/or hydrogen as growth substrates for *G. sulfurreducens*. Even when formate (Speers and Reguera, 2012) or hydrogen (Coppi *et al*., 2004) was used as electron donor, acetate was always added as the carbon source.

**Fig 2:**
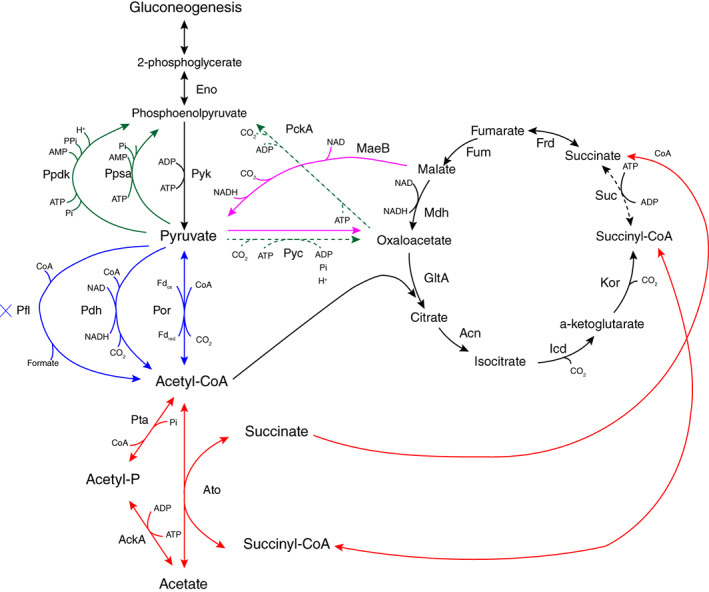
An overview of enzymes and pathways of the central metabolic network of *Geobacter sulfurreducens*; acetate activation (red arrows), CAC, conversion of pyruvate to acetyl‐CoA (blue arrows), phosphoenolpyruvate (PEP) synthase (green arrows) and anaplerotic reactions (using PEP carboxykinase and pyruvate carboxylase) (dashed green arrows). The full names of the involved proteins are included in the legend of Fig. 3. Figure is adapted after Segura *et al*., 2008.

In the central metabolic network of *G. sulfurreducens*, catabolic pathways consist of acetate uptake, acetate activation, the CAC, conversion of pyruvate to acetyl‐CoA, and anabolic pathways consist of phosphoenolpyruvate (PEP) synthase and anaplerotic reactions (using PEP carboxykinase and pyruvate carboxylase; Segura *et al*., 2008; Fig. 2).

Acetate uptake by *G. sulfurreducens* is mediated by four sodium/solute symporter proteins (AplA; GSU1068, AplB; GSU1070, AplC;GSU2352 and AplD; GSU0518). Three of these proteins, AplA, AplB and AplC, are in a tight phylogenetic cluster (Group I) and presence of at least two of them is necessary for acetate uptake (Risso *et al*., 2008; Mahadevan *et al*., 2011). The fourth protein, AplD, is phylogenetically distinct (Group II) and its function is independent of acetate availability (Risso *et al*., 2008). When growth is limited by acetate availability, Group I proteins have shown to be highly transcribed (Risso *et al*., 2008).

**Fig 3:**
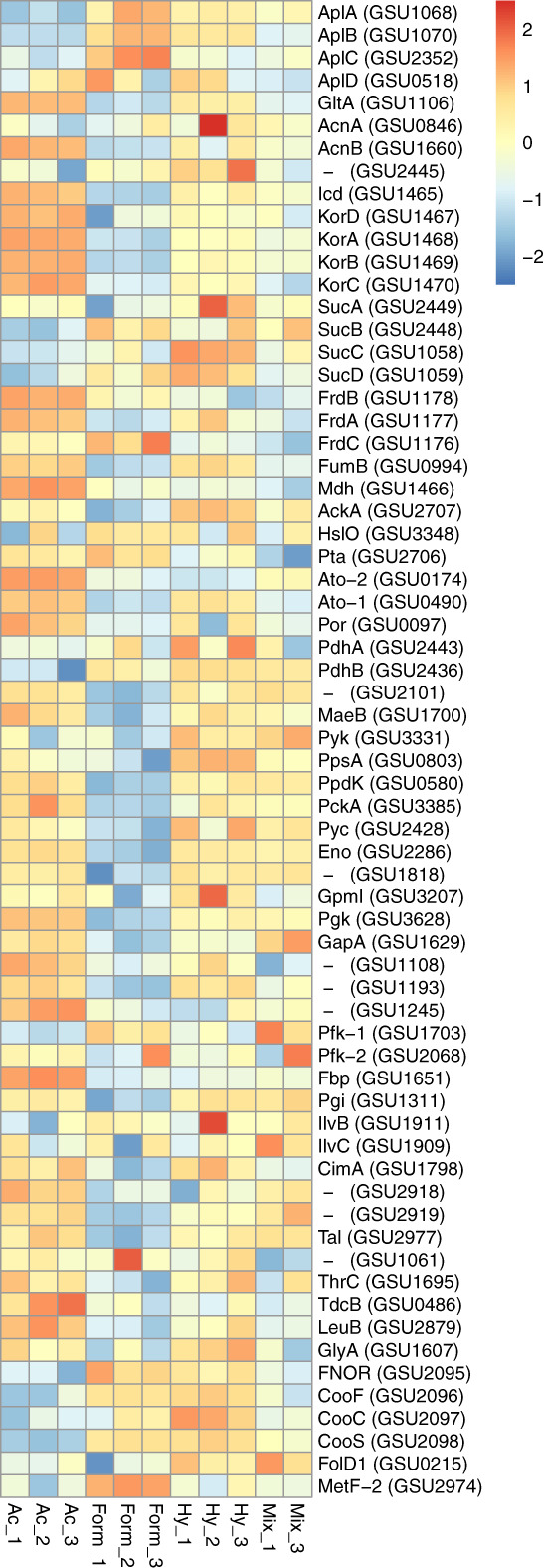
Relative abundance of the detected proteins in the central metabolic network of *Geobacter sulfurreducens*. Protein abundance levels are shown after Z‐score normalization. The colour intensity indicates the degree of protein presence, where high relative abundance is indicated in red and low relative abundance in blue. The rows in the heat map show the detected proteins in four different growth conditions. The columns show the electron donors used by *G. sulfurreducens* organized from left to right as formate, acetate, hydrogen and mix (containing all three electron donors). The abbreviation of the proteins are: sodium/solute symporter family protein (AplA, AplB, AplC, AplD), citrate synthase (GltA), aconitate hydratase 1 (AcnA), aconitate hydratase 2 (AcnB), aconitate hydratase, putative (GSU2445), isocitrate dehydrogenase, NADP‐dependent (Icd), 2‐oxoglutarate:ferredoxin oxidoreductase, ferredoxin subunit (KorD), 2‐oxoglutarate:ferredoxin oxidoreductase, alpha subunit (KorA), 2‐oxoglutarate:ferredoxin oxidoreductase, thiamin diphosphate binding subunit (KorB), 2‐oxoglutarate:ferredoxin oxidoreductase, gamma subunit (KorC), 2‐oxoglutarate dehydrogenase, E1 protein (SucA), 2‐oxoglutarate dehydrogenase, E2 protein, dihydrolipoamide succinyltransferase (SucB), succinyl‐CoA synthetase, beta subunit (SucC), succinyl‐CoA synthetase, alpha subunit (SucD), succinate dehydrogenase/fumarate reductase, iron–sulfur protein (FrdB), succinate dehydrogenase/fumarate reductase, flavoprotein subunit (FrdA), succinate dehydrogenase/fumarate reductase, cytochrome b558 subunit (FrdC), fumarate hydratase, class I (FumB), malate dehydrogenase, NAD‐dependent (Mdh), acetate kinase (AckA), chaperonin Hsp33 (HslO), phosphate acetyltransferase (Pta), succinyl:acetate coenzyme A transferase (Ato‐2), succinyl:acetate coenzyme A transferase (Ato‐1), pyruvate:ferredoxin/flavodoxin oxidoreductase (Por), pyruvate dehydrogenase E1 component subunit alpha (PdhA), pyruvate dehydrogenase E1 component subunit beta (PdhB), formate acetyltransferase/ glycerol dehydratase, putative (GSU2101), NADP‐dependent malic enzyme (MaeB), pyruvate kinase (Pyk), phosphoenolpyruvate synthase (PpsA), pyruvate phosphate dikinase (PpdK), phosphoenolpyruvate carboxykinase, GTP‐dependent (PckA), pyruvate carboxylase (Pyc), enolase (Eno), phosphoglycerate mutase family protein (GSU1818), phosphoglycerate mutase, 2,3‐bisphosphoglycerate‐independent (GpmI), phosphoglycerate kinase (Pgk), glyceraldehyde‐3‐phosphate dehydrogenase, type I (GapA), aldehyde dehydrogenase (GSU1108), ketose‐1,6‐bisphosphate aldolase, class II, putative (GSU1193), ketose‐1,6‐bisphosphate aldolase, class II, putative (GSU1245), 6‐phosphofructokinase (Pfk‐1), 6‐phosphofructokinase, ATP‐dependent (Pfk‐2), fructose‐1,6‐bisphosphatase (Fbp), glucose‐6‐phosphate isomerase (Pgi), acetolactate synthase (IlvB), ketol‐acid reductoisomerase (IlvC), 2‐isopropylmalate synthase (CimA), triose‐phosphate isomerase (GSU1628), transketolase, C‐terminal subunit (GSU2918), transketolase, N‐terminal subunit (GSU2919), transaldolase (Tal), aspartate transaminase (GSU1061), threonine synthase (ThrC), (TdcB), 3‐isopropylmalate dehydrogenase (LeuB), (GlyA), NADH oxidase, putative or FAD‐dependent pyridine nucleotide‐disulfide oxidoreductase family protein (FNOR), carbon monoxide dehydrogenase‐associated iron–sulfur cluster binding oxidoreductase (CooF), carbon monoxide dehydrogenase accessory protein (CooC), carbon monoxide dehydrogenase, catalytic subunit (CooS), sensory box protein (RcoM), bifunctional protein FolD 1 (FolD1), 5‐methyltetrahydrofolate—homocysteine S‐methyltransferase and 5,10‐methylenetetrahydrofolate reductase (MetF‐2).

Interestingly, when we compared the abundance of AplA, AplB and AplC between the different growth conditions to the acetate condition, we found significantly higher presence of these proteins under formate (12.8, 19.1, 9.7 fold, respectively) and hydrogen conditions (9.7, 15.3, 1.6 fold, respectively) as opposed to the mix condition (6.2, 0.9, 0.4 fold, respectively; Fig. 3). In line with the former studies (Risso *et al*., 2008), AplD showed almost similar abundance in formate (0.1), hydrogen (0.9), and mix condition (−4.4) compared to the acetate condition (Fig. 3).

Following uptake, acetate is activated to acetyl‐CoA for oxidation through the CAC and for gluconeogenesis. Acetate activating proteins are succinyl CoA:acetate CoA‐transferase (Ato‐1; GSU0490, Ato‐2; GSU0174), acetate kinase (AckA; GSU2707), and phosphotransacetylase (Pta; GSU2706; Segura *et al*., 2008; Fig. 2, red arrows). In all the conditions with or without acetate, these proteins were present, but clearly with higher abundance of Ato‐1&2 in the acetate condition, i.e., Ato‐1 (1.8 fold vs Hy, 9.9 fold vs Fo, 8.9 fold vs Mix) and Ato‐2 (30.6 fold vs Hy, 25.5 fold vs Fo, 17.3 fold vs Mix), AckA in the hydrogen (1.6 fold vs Ac, 4.3 fold vs Fo, 11 fold vs Mix) and Pta in the formate condition (0.7 fold vs Ac, 2.2 fold vs Hy, 11.4 fold vs Mix; Fig. 3). In the acetate condition, the higher abundance of Ato‐1&2 over AckA/Pta may indicate that the CAC is the main route for acetate conversion. An additional acetate activation pathway via AckA/Pta is necessary to provide enough acetyl‐CoA for pyruvate synthesis, and to fulfil gluconeogenic and anaplerotic demands. (Fig. 2, red arrows; Segura *et al*., 2008).

Acetyl‐CoA in the oxidation route reacts with oxaloacetate and enters the CAC (Fig. 2). We detected all enzymes from the CAC (Galushko and Schink, 2000) in the four growth conditions with clearly higher abundance in the acetate condition (Fig. 3). For instance, citrate synthase (CS; GltA; GSU1106) that is needed for the entry of acetyl‐CoA into the CAC showed significantly the highest abundance in the acetate condition (3.5 fold vs Hy, 11.3 fold vs Fo, 15.3 fold vs Mix; Fig. 3). CS is directly correlated with acetate oxidation in the CAC and has been considered as a key marker for metabolic rates of *G. sulfurreducens* in the environment (Holmes *et al*., 2005; Wilkins *et al*., 2011). A recent study showed that during chemolithoautotrophic growth of *G. sulfurreducens*, citrate synthase can perform the necessary cleavage of citrate for the reversed oxidative CAC to grow on formate or hydrogen and CO_2_ (Zhang *et al*., 2020). Presence of citrate synthase in hydrogen and formate conditions in our study indicates the reductive function of CS in roCAC.

Another key enzyme of the CAC is succinyl‐CoA synthetase that reversibly catalyses succinate to succinyl‐CoA in the CAC (Fig. 2). Interestingly, the four subunits of this enzyme (SucA; GSU2449, SucB; GSU2448, SucC; GSU1058, SucD; GSU1059) were more abundant in the hydrogen (2.5, 3.8, 4.8, 6.8 fold, respectively), formate (−2.3, 5.7, 1.2, 4.8 fold, respectively) compared to the acetate containing conditions (Fig. 3). In contrast, Ato‐1 and Ato‐2 were more abundant in the acetate condition (Fig. 3). Notably, Ato‐1&2, which activate acetate, have an additional function of converting succinyl‐CoA to succinate (Fig. 2, red arrows). Therefore, rather than succinyl‐CoA synthetase, Ato‐1&2 seem to be the main producers of succinate in the acetate condition. In line with this, a former study showed that when acetate was used by *G. sulfurreducens*, succinyl‐CoA synthetase activity was reduced and succinate was formed in a transferase reaction with acetate as the CoA‐acceptor (Galushko and Schink, 2000). Another study showed that a *G. sulfurreducens* mutant lacking Ato activity could not grow with acetate alone, and grew only when hydrogen was added as electron donor (Segura *et al*., 2008). These findings indicate that when acetate alone is used, Ato‐1&2 fulfil the function of succinyl‐CoA synthetase. In formate‐adapted *G. sulfurreducens*, deletion of the succinyl‐CoA synthetase genes prevented growth with formate as substrate (Zhang *et al*., 2020), which indicated that the absence of succinyl‐CoA synthetase disrupts carbon assimilation via roCAC in chemolithoautotrophic growth by *G. sulfurreducens*. GSU0514 is a transcriptional regulator of the IclR family involved in the repression of the transcription of *sucC* and *sucD* (Summers *et al*., 2012). A GSU0514‐dependent regulatory mechanism on succinyl‐CoA synthetase was shown when *G. sulfurreducens* was adapted to acetate, formate or lactate (Summers *et al*., 2012; Zhang *et al*., 2020). However, in our study both GSU0514 (0.87 vs Ac, 2.5 vs Fo, 6.6 vs Mix) and succinyl‐CoA synthetase were more abundant in the hydrogen condition. This challenges the proposed regulatory role of GSU0514 when hydrogen was used as electron donor and merits further investigation.

Another notable finding of our study was the low abundance of proteins involved in acetate metabolism in the mix condition (Fig. 3). This indicates that in the presence of hydrogen and formate, acetate was mostly used as a carbon source for cell synthesis, which is in line with the former reports on reduced acetate oxidation in the CAC in the presence of hydrogen (Ueki and Lovley, 2010).

In the route of converting malate to oxaloacetate via pyruvate (anaplerosis), we detected pyruvate carboxylase (Pyc; GSU2428) and the NAD‐dependent malic enzyme (MaeB; GSU1700; Fig. 3; Fig. 2, purple arrows). This pathway could potentially be an alternative for the malate dehydrogenase (Mdh), but at the cost of an extra ATP by Pyc (Segura *et al*., 2008). We detected both malate dehydrogenase and malic enzyme in all four growth conditions (Fig. 3). However, malate dehydrogenase (Mdh; GSU1466) showed significantly higher abundance in the acetate condition (7 fold vs Hy, 6.4 fold vs Fo, 14.6 fold vs Mix; Fig. 3). Similarly, malic enzyme (MaeB) was also higher in the acetate condition (0.8 fold vs Hy,4.2 fold vs Fo, 1 fold vs Mix) (Fig. 3). This indicates that (i) even in presence of Mdh, the anaplerotic pathway with an extra ATP cost is still necessary and (ii) more oxaloacetate via anaplerosis needs to be routed to the citrate synthase reaction to replenish CAC intermediates or to the gluconeogenesis for PEP synthase. A previous study reported that Mdh‐deficient *G. sulfurreducens* strain was able to grow with hydrogen as the electron donor, but not with acetate (Segura *et al*., 2008) which indicates MaeB is mostly functional in anabolism.

We further evaluated pyruvate oxidation to acetyl‐CoA with the three predicted reactions (Fig. 2, blue arrows) under the studied growth conditions. Having these three pathways in *G. sulfurreducens* seem to give a versatile approach to capture energy and carbon (Methe *et al*., 2003). The pyruvate: ferredoxin/flavodoxin oxidoreductase (Por; GSU0097) encoding a putative homodimeric type enzyme was abundant in the acetate condition (2.8 fold vs Hy, 4.1 fold vs Fo, 2.7 fold vs Mix). The alternative reactions using pyruvate dehydrogenase (PdhA; GSU2443, PdhB; GSU2436) were present in all the conditions but were the highest in the hydrogen condition i.e. PdhA (3.6 fold vs Ac, 2.7 fold vs Fo, 3.9 fold vs Mix) and PdhB (6.6 fold vs Ac, 1.8 fold vs Fo, 2.9 fold vs Mix), and were the lowest in acetate condition (Fig. 3). Pyruvate formate‐lyase enzyme (Pfl; GSU2102) was not detected in any of the conditions, and does not seem to be functional under the studied growth conditions.

Acetyl‐CoA to pyruvate conversion by pyruvate oxidoreductase (Por) has also been proposed to initiate gluconeogenesis in *G. sulfurreducens* grown with Fe(III) (Yang *et al*., 2010). According to a previous study, a POR mutant strain did not grow with acetate as the carbon source and electron donor but grew with acetate and pyruvate (Segura *et al*., 2008). This indicated that Por has an essential role in *G. sulfurreducens* to assimilate acetate. In line with this, in our study high abundance of Por was detected in the acetate condition (Fig. 3).

One key step in the gluconeogenesis is the synthesis of phosphoenolpyruvate (PEP). PEP synthesis via pyruvate can occur through three pathways with different energetic demands (Fig. 2, green, and green dashed arrows); pyruvate phosphate dikinase (PpdK; GSU0580), PEP synthase (PpsA; GSU0803) and anaplerotic reactions by pyruvate carboxylase (Pyc; GSU2428) and phosphoenolpyruvate carboxykinase, GTP‐dependent (PckA; GSU3385). Variation in electron acceptors (Fe(III) or fumarate) has been reported to influence the contribution of different pathways in PEP synthesis (Yang *et al*., 2010). For instance, in Fe(III)‐grown cultures, PEP was mainly generated from pyruvate via PpsA/PpdK compared to fumarate‐grown cultures where PEP synthesis from oxaloacetate by PckA was the major route. The Ppdk pathway is energetically more favourable than either the PckA or the PpsA pathway. This is because the Ppdk reaction produces diphosphate, which is hydrolyzed to translocate protons across the cell membrane, resulting in an energetic advantage (Segura *et al*., 2008). This is in general favourable for the growth with Fe(III) citrate as electron acceptor because of the already low energy yields compared to fumarate as the electron acceptor. In our study using Fe(III), the abundance of PpdK, PpsA, and Pyc and PckA varied with different electron donors (Fig. 3). The higher abundance of PpdK was found in the acetate containing condition (1.3 fold vs Hy, 8.3 fold vs Fo, 0.0 fold vs Mix), PpsA in the hydrogen condition (6.1 fold vs Ac, 11.7 fold vs Fo, 4.9 fold vs Mix), and Pyc (from anaplerotic pathway) in the hydrogen condition (1.1 fold vs Ac, 4.8 fold vs Fo, − 0.2 fold vs Mix) and PckA in the acetate condition (2.3 fold vs Hy, 6.4 fold vs Fo, 2.2 fold vs Mix; Fig. 3). This high contribution of PckA to gluconeogenesis in the acetate cultures seems to decreases the oxaloacetate level. This seems necessary as the conversion of malate to oxaloacetate by malate dehydrogenase is thermodynamically unfavourable (standard free energy change is +29.7 kJ/mol; Segura *et al*., 2008).

It was proposed that the electrons from hydrogen directly enter the menaquinone pool, and that this avoids the energy demand via the CAC (Segura *et al*., 2008). However, presence of the CAC enzymes in the hydrogen culture (although with reduced activities compared to the acetate culture) contribute to anabolism via roCAC. Our results in the hydrogen condition show that gluconeogenesis may initiate from pyruvate by Ppsa or/and oxaloacetate by PckA. Possibly decreased energy conservation via the CAC in the presence of hydrogen may make intermediates such as oxaloacetate more available for anabolic purposes such as amino acid biosynthesis (Yang *et al*., 2010; Zhang *et al*., 2020). Acetyl‐CoA may replenish from phosphoenolpyruvate via Pyk, and subsequently from pyruvate by Pdh or Por. Pyruvate kinase (Pyk; GSU3331) that converts phosphoenolpyruvate (PEP) to pyruvate was more abundant in the hydrogen condition (1.1 fold vs Ac, 1.4 fold vs Fo, 5.1 fold vs Mix; Fig. 3). Overall, formate‐grown cells in our study showed the lowest abundance of enzymes involved in the CAC and PEP synthesis pathways compared to the other conditions.

### Hydrogenases and formate dehydrogenases in response to electron donor changes

*G. sulfurreducens* has been shown to produce and consume hydrogen (Caccavo *et al*., 1994; Coppi *et al*., 2004). Four [NiFe]‐hydrogenases (Hya, Hyb, Hox and Mvh cluster), and three other hydrogenases clusters (Hyp, Ehr and Hdr clusters) have been identified in the genome of *G. sulfurreducens* (Methe *et al*., 2003; Coppi *et al*., 2004).

**Fig 4:**
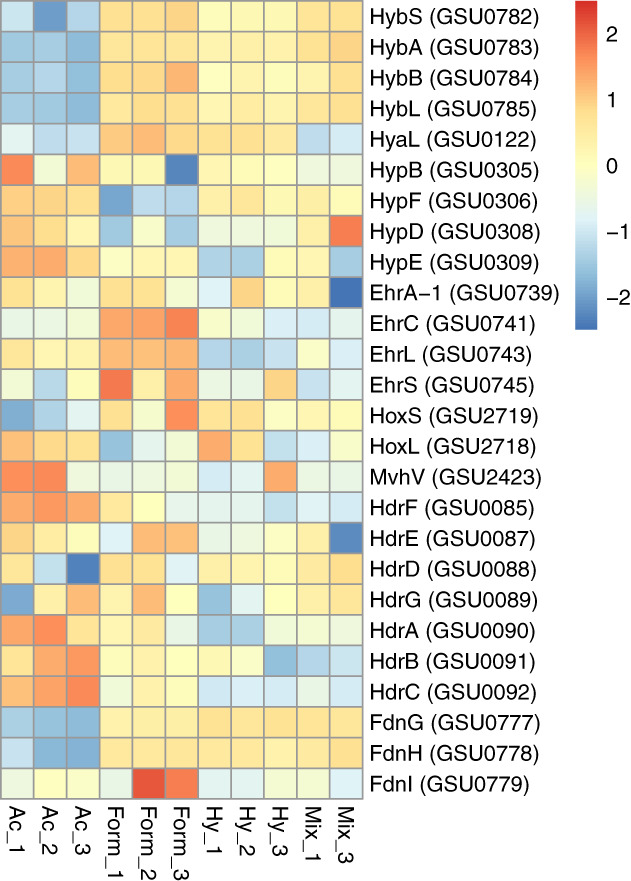
Relative abundance of the detected hydrogenases. Protein abundance levels are shown after Z‐score normalization. The colour intensity indicates the degree of protein presence, where high relative abundance is indicated in red and low relative abundance in blue. The rows in the heat map show the detected proteins in four different growth conditions. The columns show the electron donors used by *Geobacter sulfurreducens* organized from left to right as formate, acetate, hydrogen and mix (containing all three electron donors). The abbreviation of the proteins are: Hyb: periplasmically oriented, membrane‐bound [NiFe]‐hydrogenase, small subunit (HybS), periplasmically oriented, membrane‐bound [NiFe]‐hydrogenase, iron–sulfur cluster binding subunit (HybA), periplasmically oriented, membrane‐bound [NiFe]‐hydrogenase, cytochrome b subunit (HybB), periplasmically oriented, membrane‐bound [NiFe]‐hydrogenase, large subunit (HybL). Hya: periplasmically oriented, membrane‐bound [NiFe]‐hydrogenase, large subunit (HyaL). Hyp: hydrogenase accessory protein HypB, hydrogenase maturation protein HypF, hydrogenase expression/formation protein HypD, hydrogenase expression/formation protein HypE. Ehr: Ech‐hydrogenase‐related complex, NuoL‐like integral membrane subunit (EhrA‐1), Ech‐hydrogenase‐related complex, HyfE‐like integral membrane subunit (EhrC), Ech‐hydrogenase‐related complex, large subunit (EhrL), Ech‐hydrogenase‐related complex, small subunit (EhrS). Hox: bidirectional NAD‐reducing hydrogenase, small subunit (HoxS), bidirectional NAD‐reducing hydrogenase, large subunit (HoxL). Mvh: methyl‐accepting chemotaxis sensory transducer (MvhV). Hdr: heterodisulfide oxidoreductase, NAD(P)H oxidoreductase subunit F (HdrF), heterodisulfide oxidoreductase, iron–sulfur cluster binding subunit E (HdrE), heterodisulfide oxidoreductase, iron–sulfur cluster binding subunit D (HdrD), heterodisulfide oxidoreductase, iron–sulfur cluster binding subunit G (HdrG), heterodisulfide oxidoreductase, FAD binding and iron–sulfur cluster binding subunit A (HdrA), heterodisulfide oxidoreductase subunit B (HdrB), heterodisulfide oxidoreductase, iron–sulfur cluster binding subunit C (HdrC), Periplasmically oriented, membrane‐bound formate dehydrogenase, major subunit, selenocysteine‐containing (FdnG), periplasmically oriented, membrane‐bound formate dehydrogenase, iron–sulfur cluster binding subunit (FdnH), periplasmically oriented, membrane‐bound formate dehydrogenase, cytochrome b subunit (FdnI).

Previous studies on the role of the different hydrogenases in *G. sulfurreducens* have established the importance of Hyb as the only respiratory uptake hydrogenase required for growth of *G. sulfurreducens* with hydrogen as the electron donor and either fumarate, AQDS or Fe(III)‐citrate as the electron acceptor (Coppi *et al*., 2004). Moreover, Hyb is the only hydrogenase present in the *G. sulfurreducens* genome that is absent from the genome of a related species, *G. metallireducens*, which is unable to grow using hydrogen as electron donor (Lovley *et al*., 1993). As a membrane‐bound respiratory hydrogenase with a periplasmic‐oriented active site, Hyb was suggested to transfer electrons from hydrogen to the menaquinone pool, where they can be redistributed to various reductases (Coppi *et al*., 2004). Hyb is encoded by the HybSABLP (GSU0782, GSU0783, GSU0784, GSU0785 and GSU0786) operon of which we detected four subunits (GSU0782‐85) in all growth conditions (Fig. 4). Remarkably, Hyb subunits were more abundant in formate grown cells (Fig. 4). For instance, the large subunit of the uptake hydrogenase (HybL; GSU0785) showed the highest abundance in the formate condition (4.7 fold vs Hy, 25.6 fold vs Ac, 0.7 fold vs Mix). The unexpected high abundance of Hyb in the formate condition may indicate hydrogen formation from formate by *G. sulfurreducens*, which to our knowledge, has gone unnoticed in previous reports. Formate‐based hydrogen formation has been reported in diverse microorganisms including *Acetobacterium woodii* (Schuchmann and Müller, 2013), enterobacteria (Sawers, 2005), *Clostridiaceae* or *Archaea* such as *Methanococcus* (Calusinska *et al*., 2010), and the thermophilic bacterium *Thermococcus onnurineus* (Kim *et al*., 2010; Lim *et al*., 2014).

The other three hydrogenases of *G. sulfurreducens*, Hya, Hox and Mvh have not been suggested to play a direct role in hydrogen consumption, but in hydrogen production (Coppi *et al*., 2004; Tremblay and Lovley, 2012). Hya (GSU0120‐23) might be involved in converting excess reducing equivalents in the menaquinone pool to hydrogen (Tamagnini *et al*., 2002; Coppi *et al*., 2004). We only detected the large subunit, HyaL (GSU0122), that showed significantly the highest abundance in the formate condition (2.6 fold vs Hy, 15.6 fold vs Ac, 14.7 fold vs Mix). This may further indicate hydrogen formation from formate.

Although *G. sulfurreducens* is capable of hydrogen production, hydrogen‐evolving Ech hydrogenases are absent in its genome. Instead, it contains a cluster encoding a multimeric membrane‐bound Ech hydrogenase related (Ehr) complex. This gene cluster is most similar in composition to the operons encoding Ech hydrogenases. We detected the high abundance of all subunits of the Ehr complex (GSU0739‐45) in the formate condition compared to the hydrogen‐containing conditions. The physiological role of the Ehr complexes has never been investigated in *G. sulfurreducens*. However, an evolutionary link between the Ehr complexes and Ech hydrogenases that are associated with formate hydrogen lyases (Fhl) was suggested (Coppi *et al*., 2004). As Fhl is absent in the genome of *G. sulfurreducens*, the increased abundance of Ehr subunits in the formate condition in our results may support the functional link between the Ehr complex and formate dehydrogenases that allow production of hydrogen from formate.

The genome of *G. sulfurreducens* contains a cluster encoding a periplasmically oriented membrane‐bound formate dehydrogenase (Methe *et al*., 2003). Of the four subunits of formate dehydrogenase (GSU0777‐80), we detected three subunits among all the conditions (Fig. 4), but we did not find FdhD/mobA‐2 (GSU0780). The catalytic subunit of formate dehydrogenase, FdnG (GSU0777), was significantly more abundant in the hydrogen condition (5.3 fold vs Fo, 38.8 fold vs Ac, 0.01 fold vs Mix; Fig. 4). In contrast, in the formate condition, FdnH (GSU0778; 1.3 fold vs Hy, 25.9 fold vs Ac, 5.3 fold vs Mix) and FdnI (GSU0779; 14.3 fold vs Hy, 11.1 fold vs Ac, 12.7 fold vs Mix) were more abundant (Fig. 4). The observed difference in the abundance of FdhG, FdhH and FdhI in the studied growth conditions (Fig. 4) may suggest different roles for these subunits of formate dehydrogenases. For instance, FdhI is a membrane‐associated domain of CbcL (c‐ and b‐type cytochrome for low potential) that can function as a MQ oxidoreductase (Reguera and Kashefi, 2019).

Transient trace formate concentrations (0.1–0.3 mM) were detected in the different replicates of the hydrogen condition (data not shown) and the high abundance of two important subunits of formate dehydrogenase (FdnG and FdnH) indicate formate formation in the hydrogen condition. The production of formate from hydrogen and vice versa and the absence of formate hydrogen lyase (Fhl) in *G. sulfurreducens* genome might indicate the formation of formate hydrogen lyase complex to fulfil the role of the missing Fhl. This has also been suggested for anaerobes such as sulfate reducers and methanogens (Lupa *et al*., 2008; Martins and Pereira, 2013; Martins *et al*., 2015).

### Variability of electron transport proteins in response to electron donor changes

During the oxidation of acetate, formate or/nd hydrogen, electrons should be transported to the electron acceptor using specialized respiratory chains. In all the cases and according to the detected proteins in this study, produced electrons flow to menaquinone (MQ), *c*‐type cytochromes of the inner membrane (MacA, Cbc complexes; putative menaquinol oxidoreductase), periplasmic *c*‐type cytochromes (PpcA, PpcB), outer membrane cytochromes (OmcB and OmcS), and finally to Fe(III). From oxidation of acetate using the CAC, electrons and protons are carried to menaquinone in the form of NADH. The oxidation of formate and hydrogen likely directly contributes to the proton motive force and electron flow to the MQ pool in the inner membrane.

**Fig 5:**
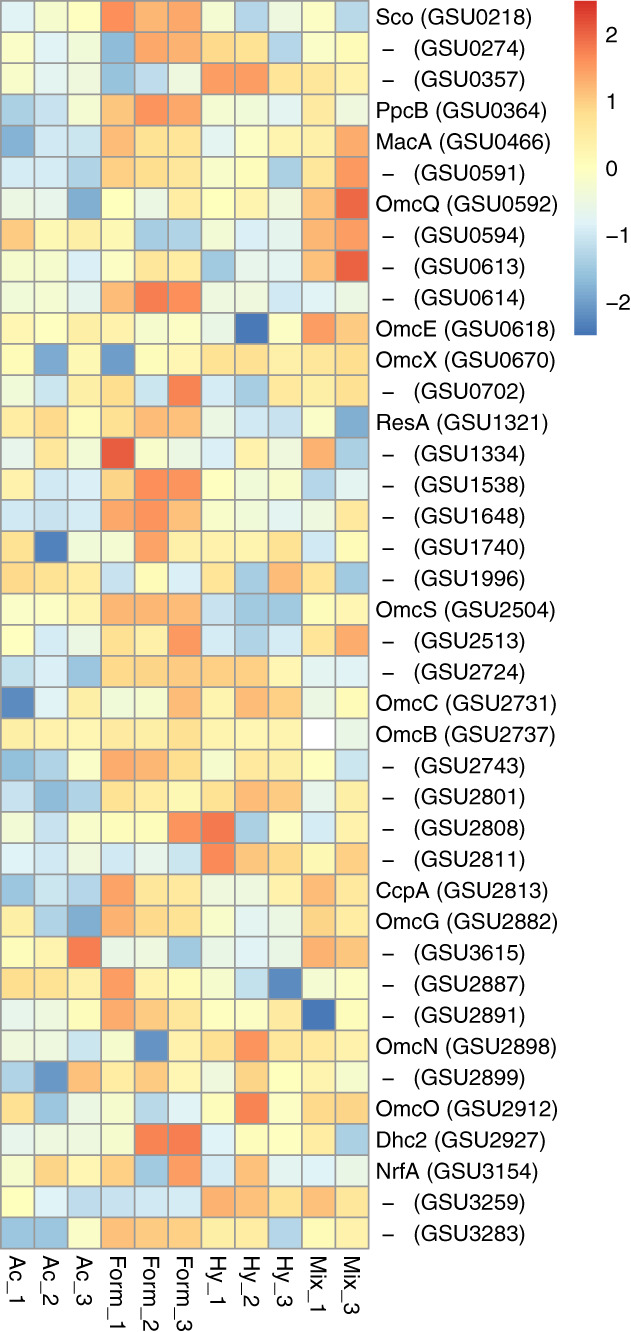
Abundance levels of the detected *C*‐type cytochrome of *Geobacter sulfurreducens*. Protein abundance levels are shown after Z‐score normalization. The colour intensity indicates the degree of protein presence, where high relative abundance is indicated in red and low relative abundance in blue. The rows in the heat map show the detected proteins in four different growth conditions. The columns show the electron donors used by *G. sulfurreducens* organized from left to right as formate, acetate, hydrogen and mix (containing all three electron donors). Protein abbreviations are: cytochrome c oxidase, coo3‐type, synthesis factor (Sco), cytochrome c/cytochrome b (GSU0274), cytochrome c nitrite reductase (GSU0357), cytochrome c (PpcB), cytochrome c peroxidase (MacA), cytochrome c (GSU0591), lipoprotein cytochrome c (OmcQ), cytochrome c (GSU0594), ResB‐like family cytochrome c biogenesis protein (GSU0613), ResC/HemX‐like cytochrome c biogenesis membrane protein (GSU0614), cytochrome c (OmcE), lipoprotein cytochrome c (OmcX), lipoprotein cytochrome c (GSU0702), apocytochrome c disulfide reductase lipoprotein ResA (ResA), cytochrome c (GSU1334), cytochrome c (GSU1538), cytochrome c (GSU1648), cytochrome c, 1 heme binding site (GSU1740), cytochrome c (GSU1996), cytochrome c (OmcS), lipoprotein cytochrome c, 1 heme binding site (GSU2513), cytochrome c (GSU2724), lipoprotein cytochrome c (OmcC), lipoprotein cytochrome c (OmcB), cytochrome c, 1 heme binding site (GSU2743), cytochrome c (GSU2801), lipoprotein cytochrome c (GSU2808), cytochrome c (GSU2811), cytochrome c peroxidase (CcpA), cytochrome c (OmcG), cytochrome c (GSU3615), lipoprotein cytochrome c (GSU2887), ResB‐like family cytochrome c biogenesis protein (GSU2891), lipoprotein cytochrome c (OmcN), lipoprotein cytochrome c (GSU2899), cytochrome c (OmcO), cytochrome c (Dhc2), cytochrome c nitrite and sulfite reductase, catalytic subunit lipoprotein (NrfA), cytochrome c (GSU3259), ResC/HemX‐like cytochrome c biogenesis membrane protein (GSU3283).

We noted that the electron transport proteins show variation in response to the electron donor changes (Fig. 5). For instance, Nuo‐1 cluster (GSU0338‐351) which encodes one of the two NADH dehydrogenase complexes clearly showed the highest abundance in the acetate condition and lowest in the formate condition (Supporting Information, Fig. S3), indicating efficient electron transfer from NADH produced by the CAC to menaquinone, which in turn transfers electrons to the terminal electron acceptor Fe(III). Proton pumping by NADH dehydrogenase complexes generates the transmembrane proton gradient that drives ATP synthesis by the ATPase complex. In all the studied conditions, we detected eight subunits of ATP synthase (GSU0108‐114 and GSU0333) with generally higher abundance in the formate and mix conditions (Supporting Information Fig. S3).

MacA (GSU0466), an inner membrane *c*‐type cytochrome was reported to be abundant during growth on Fe(III) oxide (Lloyd *et al*., 2003; Butler *et al*., 2004; Kim and Lovley, 2008). We detected MacA during growth on Fe(III) citrate with higher abundance in the formate condition (2.1 fold vs Hy, 4.4 fold vs Ac, 6.4 fold vs Mix; Fig. 5). This may indicate cytoplasmic oxidation of formate and extracellular electron transport from the inner membrane.

The genome of *G. sulfurreducens* was predicted to encode five putative menaquinol oxidoreductase protein complexes (Cbc1, Cbc3, Cbc4, Cbc5, Cbc6; Butler *et al*., 2010; Aklujkar *et al*., 2013). These proteins combine electron transfer to form a proton gradient across the inner membrane via either a Q loop or a Q cycle (Trumpower, 1990). According to former transcriptomic and proteomics data, Cbc‐like gene clusters in *G. sulfurreducens* are differentially expressed in response to electron acceptor availability or growth in syntrophic co‐culture with other strains (Mehta *et al*., 2005; Leang *et al*., 2010). Some putative structural subunits of Cbc1, Cbc3, Cbc4 and Cbc5 complexes were detected in our study with dynamic responses towards various electron donors. Generally, the highest abundance was found in the formate condition and the lowest in the acetate condition which may indicate a direct flow of electron from formate to the menaquinone pool (Supporting Information Fig. S3). Although Cbc complexes show a high degree of conservation among *Geobacter* genomes (Butler *et al*., 2010) and interact with other electron transfer proteins, these complexes still remain ill‐defined.

In order to transfer electrons across the periplasm from electron donors such as acetate that are metabolized in the cytoplasm, Cbc proteins transfer electrons to one of several small periplasmic cytochromes (Ppc). Of the five closely related periplasmic *c*‐type cytochromes (PpcA‐E) of *G. sulfurreducens* (Methe *et al*., 2003), PpcA, PpcB, PpcD and PpcE have been detected in the former proteomic study with acetate and Fe(III) citrate (Ding *et al*., 2008). In contrast, we detected only PpcB (GSU0364) and PpcA (GSU0612). PpcB was more abundant in the formate condition (6.5 fold vs Hy, 8.2 fold vs Ac, 6.2 fold vs Mix) and PpcA in the hydrogen condition (2.8 fold vs Fo, 4.6 fold vs Ac, 8.5 fold vs Mix) (Fig. 5). In contrast, a former study showed that PpcA is not required for Fe(III) reduction with hydrogen as the electron donor when a PpcA‐mutant of *G. sulfurreducens* reduced Fe(III) with hydrogen (Lloyd *et al*., 2003). This suggests that the hydrogenases involved in hydrogen oxidation coupled to Fe(III) reduction are in the periplasm and can fulfil the function of PpcA (Lloyd *et al*., 2003). In our study, the presence of other carriers in the periplasm such as formate dehydrogenases may also fulfil the function of the missing Ppcs.

From outer membrane cytochromes, OmcS (GSU2504) as the terminal Fe(III) reductase (Mehta *et al*., 2005; Qian *et al*., 2011), is essential for Fe(III) oxide reduction by *G. sulfurreducens* (Leang *et al*., 2003; Kim *et al*., 2005; Mehta *et al*., 2005; Leang *et al*., 2010). The *omcS* gene has been reported to be upregulated during growth on Fe(III) oxide but not in cultures grown on Fe(III) citrate (Mehta *et al*., 2005), or electrodes (Holmes *et al*., 2006), nor in a coculture of *G. sulfurreducens* and *G. metallireducens* where direct interspecies electron transfer (DIET) occurs (Summers *et al*., 2010). In contrast, we detected OmcS during growth on Fe(III) citrate and with significantly high abundance in the formate condition (28.3 fold vs Hy, 13.3 fold vs Ac, 18.9 fold vs Mix; Fig. 5). Although OmcS was considered essential for DIET, but not for hydrogen interspecies transfer (HIT; Shrestha *et al*., 2013), the increased abundance of OmcS in the formate condition in our study may point towards a role of OmcS in formate interspecies electron transfer (FIT). This hypothesis can be tested in the future, e.g., by mutants of *G. sulfurreducens* that lack *omcS* in co‐cultures mediating FIT. Another outer membrane cytochromes, OmcB (GSU2737) has an important function in electron transport to Fe(III) (Leang *et al*., 2003; Mehta *et al*., 2005; Leang *et al*., 2010). The localization of OmcB suggests that electron transport to the outer membrane is necessary for outside reduction of Fe(III) by *G. sulfurreducens*. Accordingly, deletion of the *omcB* gene inhibited reduction of Fe(III) citrate and Fe(III) oxide (Leang *et al*., 2003). In line with this, in our study with Fe(III) citrate, OmcB was detected in all the conditions. The high abundancy of OmcB in the formate condition (1.3 vs Ac, 1.6 vs Hy, 10.8 vs Mix fold; Fig. 5) is in line with its poor functionality in DIET in the coculture of *G. metallireducens*‐*G. sulfurreducens* (Summers *et al*., 2010) and may imply OmcB function in IIET (Indirect interspecies electron transfer).

Despite the limited range of *C*‐type cytochromes which have been discussed here, our results showed variability in abundance of other cytochromes in *G. sulfurreducens* (Fig. 5) in response to electron donor changes in presence of Fe(III) citrate. Although, this emphasizes the importance of cytochromes for Fe(III) reduction in *G. sulfurreducens*, future research is necessary to understand the roles of these cytochromes in electron transport mechanisms.

The high abundance of the studied electron transport proteins in the formate condition in *G. sulfurreducens*, may support the ease and preference of formate for conversion over acetate and hydrogen. The results also suggest that portions of the route of electron transfer to Fe(III) citrate differ based on the electron donor type.

### Chemolithoautotrophic growth of *G. sulfurreducens*

*G. sulfurreducens* has been considered a heterotrophic bacterium as it lacks a complete set of enzymes to grow with CO_2_ or other C1 compounds such as formate as sole source of carbon (Mahadevan *et al*., 2006; Feist *et al*., 2014). In contrast, potential autotrophic growth was predicted from genomic analysis via the reductive CAC or the acetyl‐CoA pathway (Methe *et al*., 2003). In line with these genomic predictions, a recent study (Zhang *et al*., 2020) and our findings corroborate chemolithoautotrophic growth of *G. sulfurreducens* on formate or hydrogen plus CO_2_ with Fe(III) citrate as electron acceptor. *G. sulfurreducens* cannot utilize citrate as electron donor or carbon source (Galushko and Schink, 2000; Esteve‐Núñez *et al*., 2005; Coppi *et al*., 2007). Citrate was not likely used as the carbon source as its concentration was always stable throughout the experiment, and we also did not detect a citrate transporter in our proteomic results as predicted from the genome of *G. sulfurreducens* (Methe *et al*., 2003). Similar arguments for the lack of citrate consumption was also presented in a recent study where chemolithoautotrophic growth of *G. sulfurreducens* on Fe(III) citrate and formate or hydrogen plus CO_2_ was shown (Zhang *et al*., 2020). Further proof for the lack of citrate consumption in the presence of acetate by *G. sulfurreducens* was provided by carbon labelling ([U‐13C6]Fe(III)citrate) showing that Fe(III) citrate was exclusively used as an electron acceptor (Yang *et al*., 2010).

Three key enzymes are indicative of the reductive CAC: 2‐oxoglutarate:ferredoxin oxidoreductase (KorA; GSU1468, KorB; GSU1469, KorC; GSU1470, KorD; GSU1467) that catalyses the carboxylation of succinyl‐CoA to 2‐oxoglutarate, fumarate reductase (FrdA; GSU1177, FrdB; GSU1178, FrdC; GSU1176) that mediates the reduction of fumarate to succinate, and ATP citrate lyase that catalyses the ATP‐dependent cleavage of citrate to acetyl CoA and oxaloacetate (Hügler *et al*., 2005). Our results showed that KorA‐D have significantly higher abundance in the acetate condition (5.9, 4.7, 5.7, 8.6 fold vs Hy; 11.2, 10.3, 10.3, 15 fold vs Fo; 14.8, 17.3, 19.7, 18.2 fold vs mix, respectively). Similarly, FrdA‐C showed higher abundance in the acetate condition (1.5, 4, 2.8 fold vs Hy; 4.2, 2.5, 4.3 fold vs Fo; 3.4, 12.2, 12.6 fold vs mix, respectively). However, the gene cluster encoding the citrate lyase enzyme is not present in the genome of *G. sulfurreducens* suggesting that this species is not able to operate the reductive CAC. It was recently reported that the thermophilic sulfur‐reducing *Desulfurella acetivorans* has a bi‐directional citrate synthase that allows autotrophic growth (Mall *et al*., 2018). Interestingly, such a bi‐directional citrate synthase was discovered in *G. sulfurreducens*, which enables roCAC (Zhang *et al*., 2020). Moreover, succinyl‐CoA synthetase, which is not involved in heterotrophic growth of *G. sulfurreducens* with acetate would be required for carbon assimilation via roCAC in chemolithoautotrophic growth with formate or hydrogen.

Another possibility for autotrophic growth is via acetyl‐CoA pathway (Methe *et al*., 2003). However, *G. sulfurreducens* was reported to lack the acetyl‐CoA pathway due to lack of genes encoding a bifunctional enzyme carbon monoxide dehydrogenase/acetyl‐Co synthase (CODH/ACS) and formyl tetrahydrofolate synthetase (FTS; Galushko and Schink, 2000; Geelhoed *et al*., 2016). In contrast, our results imply the occurrence of some reactions of the acetyl‐CoA pathway in the hydrogen and formate conditions without the addition of acetate as a carbon source. First, we found high abundance of formate dehydrogenase in formate and hydrogen conditions (Fig. 4), which is a typical enzyme of the CO/acetyl‐CoA pathway forming acetyl‐CoA for subsequent assimilation into biomass. Second, we found a high abundance of hydrogenase complexes of Ech homologue (GSU0739‐GSU0745) and Hox (GSU2718‐GSU2722) in formate and hydrogen conditions (Fig. 4) that may be involved in reverse electron transport and fulfil the FTS function by driving CO_2_ reduction to a formyl group with subsequent attachment to the C1 carrier (Methe *et al*., 2003). Third, formate was formed from hydrogen and CO_2_. Fourth, except rcoM (GSU2099), we detected significantly higher abundance of all subunits of monofunctional CODH (CooS; GSU2098, CooC; GSU2097, CooF; GSU2096, FNOR; GSU2095) and high abundance of two other proteins (FolD1; GSU0215, MetF‐2; GSU2974) in the hydrogen and formate conditions compared to the acetate‐containing conditions (Fig. 3). However, the corrinoid enzyme (GSU2386) and methyltransferase (GSU2387) which may interact with CODH during acetyl‐CoA formation were not found in our proteome results. Future experiments will be necessary to verify autotrophic growth using the acetyl‐CoA pathway, for example using labelled (^13^C) bicarbonate to track carbon flow (Zhang *et al*., 2020), and/or using *G. sulfurreducens* lacking genes of the acetyl‐CoA pathway when grown with hydrogen or formate.

## Conclusions

The differential abundance of proteins was studied for deciphering metabolic response of *G. sulfurreducens* to different electron donors for Fe(III) reduction. The growth phenotypes and the proteomics results revealed that the physiological state of *G. sulfurreducens* is altered in response to variation of electron donors. A notable finding was the utilization of formate as a preferred electron donor for *G. sulfurreducens* over acetate and/or hydrogen, whereas most of the previous physiological and genetic studies of this bacterium have used acetate as the preferred electron donor. Moreover, interconversion of formate and hydrogen occurred. Another remarkable finding was chemolithoautotrophic growth of *G. sulfurreducens* with formate or hydrogen and CO_2_ as the sole carbon source. Our results present an important framework for future dedicated studies to verify the actual role of underlying proteins in the central metabolism including hydrogen and formate metabolism and electron transfer mechanism of *G. sulfurreducens*.

## Materials and methods

### Microorganism and cultivation

*G. sulfurreducens* strain PCA^T^ (DSM 12127) was grown under strict anoxic conditions at 35°C in 120 ml serum bottles with 50 ml of bicarbonate‐buffered medium as described previously (Stams *et al*., 1993) with N_2_/CO_2_ (80:20, v/v) as the headspace. The medium was reduced with FeCl_2_ (1.3 mM).

Acetate, formate, hydrogen or their different combinations were used as electron donor(s) and Fe(III) citrate was used as the electron acceptor (Table S1). Sodium acetate and sodium formate were added from 1 M sterile anoxic stock solutions. When hydrogen was used as the electron donor, 17.9% of the headspace was filled with pure hydrogen (18.1 KPa) to reach 10 mmol hydrogen per litre of liquid medium. *G. sulfurreducens* was adapted to each culture conditions by at least five subsequent transfers (10% v/v) with the corresponding electron donor(s). All incubations were done in triplicate. Growth was determined by quantifying depletion of electron donors and production of Fe(II).

### Analytical methods

Acetate and formate were analysed using high‐performance liquid chromatograph (HPLC) using a Dionex UHPLC system (Molenaar *et al*., 2019). Headspace hydrogen was measured by gas chromatography (GC). Headspace samples (0.2 ml) taken with a 1 ml syringe were analysed using a Compact GC 4.0 (Global Analyser Solutions, The Netherlands). A molsieve 5A column operated at 100°C coupled to a Carboxen 1010 pre‐column was used for hydrogen determination, and detection was done via a thermal conductivity detector. Fe(II) and Fe(III) were quantified with the ferrozine colorimetric method (Stookey, 1970) with absorbance at 562 nm using a U‐1500 spectrophotometer (Hitachi, Chiyoda, Tokyo, Japan).

### Proteomic analyses

#### Protein extraction and separation

*G. sulfurreducens* grown on a single electron donor (acetate, formate or hydrogen) and a mixture of all three electron donors (Table S1) was used for whole cell proteomic analyses. Cells from triplicate cultures were harvested at the end of the exponential growth phase by centrifugation at 16 000*g* for 20 min at 4°C. Cell pellets were washed twice with 20 mM Tris–HCl (pH 7.5) and stored at −80°C until further use. For protein extraction, cell pellets were resuspended in 0.5 ml SDT‐lysis buffer composed of 100 mM Tris/HCl, pH 7.5, 4% w/v sodium dodecylsulfate (SDS), SIGMAFAST™, Protease Inhibitor Cocktail Tablet (Sigma‐Aldrich, Missouri), and 0.1 M dithiothreitol (DTT). The suspension was subsequently sonicated as described previously (Rupakula *et al*., 2013). Proteins were denatured by heating at 95°C for 5 min, followed by centrifugation at 16000× g for 10 min. The samples were loaded on a 10% precast polyacrylamide gel (10% Mini‐PROTEAN® TGX™ Precast Protein Gels, Bio‐Rad) with Mini‐PROTEAN Tetra system (Bio‐Rad Laboratories B.V., Veenendaal, The Netherlands). The electrophoresis procedure was done following the precast gels manufacturer's instructions by loading 40 μl of each sample in gel slots (an empty lane left next to each loaded lane). Gels were stained with Coomassie brilliant blue R250 (Merck, Darmstadt, Germany) and scanned using a G:BOX Chemi XT4 (Syngene, Cambridge, UK). To quantify the intensity of each entire lane, the GeneSys software version 1.5.5.0 (GeneTools version 4.03.01) was used. The ratio of intensity of the lanes to the lane with the highest intensity was calculated and used to prepare series of gels that had less than 10% difference in the intensity between all lanes (Sedano‐Núñez *et al*., 2018).

#### In‐gel trypsin digestion and peptide purification

SDS‐PAGE‐separated proteins were subjected to in‐gel trypsin digestion and peptide purification with slight modifications to a protocol described previously (Rupakula *et al*., 2013). All solutions were prepared in 50 mM NH_4_HCO_3_ unless otherwise stated. Disulfide bridges of proteins in each gel were reduced with DTT solution (10 mM, pH 7.6) at 60 °C for 1 h, alkylated with iodoacetamide solution (10 mM in 100 mM Tris–HCl, pH 8.0) in the dark at room temperature for 1 h with shaking at 100 rpm. After each step, gels were rinsed three times with ultra‐pure water. Gels were cut into individual lanes and each gel lane was cut into three equal slices. Each slice was further processed to pieces of about 1 mm^3^ cubes and transferred to a 0.5 ml protein LoBind tube (Eppendorf, Hamburg, Germany).

Enzymatic digestion was done by adding 50 μl of 5 ng/μl trypsin solution (Sequencing grade modified trypsin; Promega, Madison, WI, USA) to each tube, and incubating overnight at room temperature with gentle shaking. In order to stop trypsin digestion, 10% (v/v) trifluoroacetic acid solution in H_2_O was added to the supernatant to lower the pH (<5). Peptides were concentrated using a C18 Empore membrane (Empore™ Extraction Disc, 3 M, VWR Amsterdam, the Netherlands) in StageTip columns with additional C18 column material as described previously (Rappsilber *et al*., 2007; Lu *et al*., 2011). Peptides were eluted with 50% (v/v) acetonitrile (in 0.1% formic acid in H_2_O) from the column. Finally, a SpeedVac vacuum centrifuge was used to remove the acetonitrile from the samples and to concentrate the peptides. To obtain enough volume for LC–MS/MS analysis, the samples volume were adjusted to 50 μl with 0.1% (V/V) formic acid. Samples were analysed using nano‐liquid chromatography (Proxeon EASY nLC‐MS) connected to a LTQ‐Orbitrap XL (Thermo Fisher Scientific, Bremen, Germany) mass spectrometry as described previously (Lu *et al*., 2011).

### LC–MS data analysis

The protein database of *G. sulfurreducens* PCA was downloaded from Uniprot (http://www.uniprot.org). An additional database with protein sequences of common contaminants (trypsin, human keratins and bovine serum albumin) was also included in the database search. MaxQuant v. 1.5.2.8 was used to analyse MS and MS/MS spectra. False Discovery Rate (FDR) of less than 1% were set at both peptide and protein levels. Modifications for acetylation (Protein N‐term), deamidation (N, Q) and oxidation (M) were allowed to be used for protein identification and quantification. All other quantification settings were kept default. Filtering and further bioinformatics and statistical analysis were performed with Perseus v.1.5.0.8. Proteins included in the analysis contain at least two identified peptides of which at least one is unique and at least one unmodified. Reversed hits and contaminants were filtered out. Protein groups were filtered to require three valid values in at least one experimental group. Label‐free quantification (LFQ) intensities (values normalized with respect to the total amount of protein and all of its identified peptides) were used to analyse the abundance of proteins in the fractions and further statistical comparisons among conditions. LFQ intensities were transformed to logarithmic values base 10. The non‐existing LFQ intensity values have been replaced with values obtained by applying a normal distribution down shift of 1.8 and a width of 0.3 (Perseus default values). Relative protein quantification of sample to control was conducted with PERSEUS by applying two‐sample *t* tests using the ‘log LFQ intensity’ columns obtained with an FDR threshold set to 0.05 and S0 = 1. S0 = 1 indicates that both the average protein abundance ratio and the *t* test *P* value have equal weight to decide whether the protein is significantly different between the two different growth conditions. Proteins significantly (*P* < 0.05) present under given condition were mentioned throughout the text. To determine the proteins fold change between two conditions, log protein abundance ratio of those conditions were considered. In order to make sensible ratio calculations possible, protein groups with a logarithmic LFQ intensity of zero of all treatments were deleted from the MaxQuant result table. To analyse proteins detected in each experimental group (Venn diagrams) proteins were considered when they had been detected in two samples in each experimental group. Z‐score normalization in which the mean of each row (where each row is a protein in triplicate and in different conditions) is subtracted from each value and the result divided by the standard deviation of the row was applied before clustering of the samples; Hierarchical clustering was performed using relative intensity LFQ data, Pearson's correlation as a distance metric and agglomerative hierarchical clustering. For the enrichment analysis, only proteins detected in at least two samples in each experimental group were considered. The enrichment analysis was performed using a hypergeometric function to model background probabilities as implemented in the R ‘phyper’ function. PCA, heatmaps, hierarchical trees and enrichment analysis were done using R (Team, 2016). The R package Venn Diagram was used for Venn diagrams (Chen and Boutros, 2011); ‘prcomp’ command was used for PCA, and ‘hclust’ was used for hierarchical clustering. The protein groups of *G. sulfurreducens* are listed in Supplementary Table S2. Proteins detected in our proteomic analysis were used to prepare the heatmaps.

## Supporting information


**Fig. S1.** Venn diagram of proteins detected in *G. sulfurreducens* cultures grown on four different electron donors and Fe(III) as electron acceptor.
**Fig. S2.** Principal Component Analysis (PCA) performed for *G. sulfurreducens* protein profiles obtained from each triplicate grown under four different conditions with (A) and without (B) Mix_2 .
**Fig. S3.** Relative abundance of the detected proteins in the central metabolic network of *G. sulfurreducens*. Protein abundance levels are shown after Z‐score normalization. The colour intensity indicates the degree of protein presence, where high relative abundance is indicated in red and low relative abundance in blue. The rows in the heat map show the detected proteins in four different growth conditions. The columns show the electron donors used by *G. sulfurreducens* organized from left to right as formate, acetate, hydrogen and mix (containing all three electron donors). All data are shown in triplicates expect mix condition which is shown in duplicate. The abbreviation of the proteins are: NADH dehydrogenase I, B subunit (NuoB), NADH dehydrogenase I, C subunit (NuoC), NADH dehydrogenase I, D subunit (NuoD), NADH dehydrogenase I, E subunit (NuoE‐1), NADH dehydrogenase I, F subunit (NuoF‐1), NADH dehydrogenase I, G subunit (NuoG‐1), NADH dehydrogenase I, H subunit (NuoH‐1), NADH dehydrogenase I, I subunit (NuoI‐1), NADH dehydrogenase I, J subunit (NuoJ‐1), NADH dehydrogenase I, L subunit (NuoL‐1), NADH dehydrogenase I, M subunit (NuoM‐1), NADH dehydrogenase I, B/C/D subunits (NuoBCD), NADH dehydrogenase I, I subunit (NuoI‐2), NADPH oxidoreductase, beta subunit (SfrB), NADPH oxidoreductase, alpha subunit (SfrA), Menaquinol oxidoreductase complex Cbc5, cytochrome c subunit, putative, 7 heme binding sites (CbcA), Menaquinol oxidoreductase complex Cbc5, cytochrome c subunit, putative, 12 heme binding sites (CbcC), Menaquinol oxidoreductase complex Cbc5, cytochrome c subunit, putative, HAMP domain‐containing, 2 heme binding sites (CbcD), Menaquinol oxidoreductase complex Cbc4, iron–sulfur cluster binding subunit, putative (CbcT), Menaquinol oxidoreductase complex Cbc3, iron–sulfur cluster binding subunit, putative (CbcV), Menaquinol oxidoreductase complex Cbc3, cytochrome b subunit, putative (CbcW), Menaquinol oxidoreductase complex Cbc3, cytochrome c subunit, putative, 5 heme binding sites (CbcX), Cytochrome c, 9 heme binding sites, and cytochrome b (CbcY), ATP synthase F0, B′ subunit (AtpX), ATP synthase F0, B subunit (AtpF), ATP synthase F1, delta subunit (AtpH), ATP synthase F1, alpha subunit (AtpA), ATP synthase F1, gamma subunit (AtpG), ATP synthase F1, beta subunit (AtpD), ATP synthase F1, epsilon subunit (AtpC), ATP synthase F0, C subunit (AtpE).
**Fig. S4**. Relative abundance of the detected pilin proteins of *G. sulfurreducens*. Protein abundance levels are shown after Z‐score normalization. The colour intensity indicates the degree of protein presence, where high relative abundance is indicated in red and low relative abundance in blue. The rows in the heat map show the detected proteins in four different growth conditions. The columns show the electron donors used by *G. sulfurreducens* organized from left to right as formate, acetate, hydrogen and mix (containing all three electron donors). All data are shown in triplicates expect mix condition which is shown in duplicate. Protein abbreviations are: type II secretion system pseudopilin oxpG (OxpG), pilin domain 1 protein pilA (PliA‐N), pilin domain 2 protein (PilA‐C), thiamin biosynthesis protein ThiI‐related adenine nucleotide alpha hydrolase superfamily protein (GSU0434), type IV pilus biogenesis ATPase PilB(PilB), type IV pilus inner membrane protein PilC (pilC), type IV pilus biogenesis ATPase PilM (PilM), type IV pilus biogenesis protein PilN (PilN), type IV pilus biogenesis protein PilO (PilO), type IV pilus secretin lipoprotein PilQ (PilQ), type IV pilus assembly lipoprotein PilP (PilP), sensor histidine kinase PilS (PAS, HisKA, HATPase_c) (PilS), sigma‐54‐dependent transcriptional response regulator PilR (REC, sigma54 interaction, HTH8) (PilR), twitching motility pilus retraction protein (pilT‐1), twitching motility pilus retraction protein (PilT‐4).
**Table S1**. Cultivation conditions for *G. sulfurreducens* used in this study. The growth conditions shown in bold were used for proteomic analysis.

**Table S2.** The protein groups of *Geobacter sulfurreducens*.

**Supplementary file 1.** List of detected protein of *G. sulfurreducens* and statistical analysis under different conditions.
