# Supplementary material for: Comparative proteomics of Geobacter sulfurreducens PCAT in response to acetate, formate and/or hydrogen as electron donor
Source: Environ Microbiol. 2020 Nov 20;23(1):299–315. doi: 10.1111/1462-2920.15311 (PMC7894505; doi:10.1111/1462-2920.15311)
Supplement: Supplementary file 1 — Fig. S1. Venn diagram of proteins detected in G. sulfurreducens cultures grown on four different electron donors and Fe(III) as electron acceptor. Fig. S2. Principal Component Analysis (PCA) performed for G. sulfurreducens protein profiles obtained from each triplicate grown under four different conditions with (A) and without (B) Mix_2 . Fig. S3. Relative abundance of the detected proteins in the central metabolic network of G. sulfurreducens. Protein abundance levels are shown after Z‐score normalization. The colour intensity indicates the degree of protein presence, where high relative abundance is indicated in red and low relative abundance in blue. The rows in the heat map show the detected proteins in four different growth conditions. The columns show the electron donors used by G. sulfurreducens organized from left to right as formate, acetate, hydrogen and mix (containing all three electron donors). All data are shown in triplicates expect mix condition which is shown in duplicate. The abbreviation of the proteins are: NADH dehydrogenase I, B subunit (NuoB), NADH dehydrogenase I, C subunit (NuoC), NADH dehydrogenase I, D subunit (NuoD), NADH dehydrogenase I, E subunit (NuoE‐1), NADH dehydrogenase I, F subunit (NuoF‐1), NADH dehydrogenase I, G subunit (NuoG‐1), NADH dehydrogenase I, H subunit (NuoH‐1), NADH dehydrogenase I, I subunit (NuoI‐1), NADH dehydrogenase I, J subunit (NuoJ‐1), NADH dehydrogenase I, L subunit (NuoL‐1), NADH dehydrogenase I, M subunit (NuoM‐1), NADH dehydrogenase I, B/C/D subunits (NuoBCD), NADH dehydrogenase I, I subunit (NuoI‐2), NADPH oxidoreductase, beta subunit (SfrB), NADPH oxidoreductase, alpha subunit (SfrA), Menaquinol oxidoreductase complex Cbc5, cytochrome c subunit, putative, 7 heme binding sites (CbcA), Menaquinol oxidoreductase complex Cbc5, cytochrome c subunit, putative, 12 heme binding sites (CbcC), Menaquinol oxidoreductase complex Cbc5, cytochrome c subunit, putative, HAMP domain‐containing, 2 heme binding sites (CbcD [file EMI-23-299-s001.docx]

**Supporting information**

**Comparative proteomics of *Geobacter sulfurreducens* PCA^T^ in response to acetate, formate and/or hydrogen as electron donor**

Monir Mollaei^1,2^, Peer H.A. Timmers^1,2^, Maria Suarez-Diez^3^, Sjef Boeren^4^, Antonie H. van Gelder^2^, Alfons J.M. Stams^2,5^ and Caroline M. Plugge^1,2*^

^1^Wetsus, European Centre of Excellence for Sustainable Water Technology, Oostergoweg 9, 8911MA Leeuwarden, The Netherlands.

^2^Laboratory of Microbiology, Wageningen University & Research, Stippeneng 4, 6708 WE Wageningen, The Netherlands.

^3^Laboratory of Systems and Synthetic Biology, Wageningen University & Research, Stippeneng 4, 6708 WE Wageningen, The Netherlands.

^4^Laboratory of Biochemistry, Wageningen University & Research, Stippeneng 4, 6708 WE Wageningen, The Netherlands.

^5^Centre of Biological Engineering, University of Minho, Campus de Gualtar, Braga, 4710-057, Portugal.

***Corresponding author**: Dr. Caroline Plugge. Laboratory of Microbiology, Wageningen University & Research, Stippeneng 4, 6708 WE Wageningen, The Netherlands. Tel. (+31) 317 483 752; e-mail: [caroline.plugge@wur.nl](mailto:caroline.plugge@wur.nl)

**
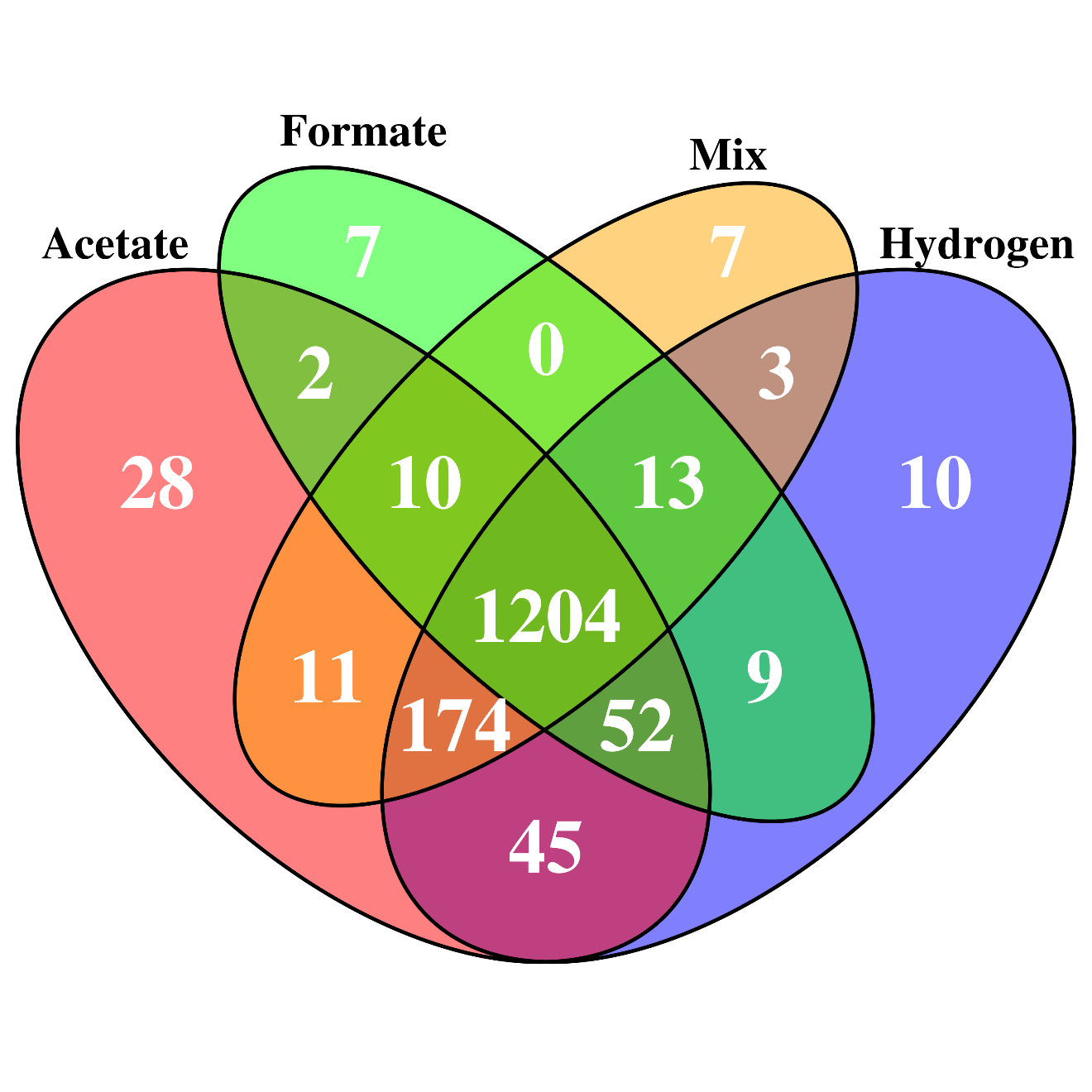
**

**Figure S1.** Venn diagram of proteins detected in *G. sulfurreducens* cultures grown on four different electron donors and Fe(III) as electron acceptor.

**Figure S2.** Principal Component Analysis (PCA) performed for *G. sulfurreducens* protein proﬁles obtained from each triplicate grown under four diﬀerent conditions with (top panel) and without (bottom panel) mix-2.

**Figure S3.** Relative abundance of the detected proteins in the central metabolic network of *G. sulfurreducens*. Protein abundance levels are shown after Z-score normalization. The colour intensity indicates the degree of protein presence, where high relative abundance is indicated in red and low relative abundance in blue. The rows in the heat map show the detected proteins in four different growth conditions. The columns show the electron donors used by *G. sulfurreducens* organized from left to right as formate, acetate, hydrogen and mix (containing all three electron donors).The abbreviation of the proteins are: NADH dehydrogenase I, B subunit (NuoB), NADH dehydrogenase I, C subunit (NuoC), NADH dehydrogenase I, D subunit (NuoD), NADH dehydrogenase I, E subunit (NuoE-1), NADH dehydrogenase I, F subunit (NuoF-1), NADH dehydrogenase I, G subunit (NuoG-1), NADH dehydrogenase I, H subunit (NuoH-1), NADH dehydrogenase I, I subunit (NuoI-1), NADH dehydrogenase I, J subunit (NuoJ-1), NADH dehydrogenase I, L subunit (NuoL-1), NADH dehydrogenase I, M subunit (NuoM-1), NADH dehydrogenase I, B/C/D subunits (NuoBCD), NADH dehydrogenase I, I subunit (NuoI-2), NADPH oxidoreductase, beta subunit (SfrB), NADPH oxidoreductase, alpha subunit (SfrA), Menaquinol oxidoreductase complex Cbc5, cytochrome c subunit, putative, 7 heme-binding sites (CbcA), Menaquinol oxidoreductase complex Cbc5, cytochrome c subunit, putative, 12 heme-binding sites (CbcC), Menaquinol oxidoreductase complex Cbc5, cytochrome c subunit, putative, HAMP domain-containing, 2 heme-binding sites (CbcD), Menaquinol oxidoreductase complex Cbc4, iron-sulfur cluster-binding subunit, putative (CbcT), Menaquinol oxidoreductase complex Cbc3, iron-sulfur cluster-binding subunit, putative (CbcV), Menaquinol oxidoreductase complex Cbc3, cytochrome b subunit, putative (CbcW), Menaquinol oxidoreductase complex Cbc3, cytochrome c subunit, putative, 5 heme-binding sites (CbcX), Cytochrome c, 9 heme-binding sites, and cytochrome b (CbcY), ATP synthase F0, B' subunit (AtpX), ATP synthase F0, B subunit (AtpF), ATP synthase F1, delta subunit (AtpH), ATP synthase F1, alpha subunit (AtpA), ATP synthase F1, gamma subunit (AtpG), ATP synthase F1, beta subunit (AtpD), ATP synthase F1, epsilon subunit (AtpC), ATP synthase F0, C subunit (AtpE).

**Figure S4**. Relative abundance of the detected pilin proteins of *G. sulfurreducens*. Protein abundance levels are shown after Z-score normalization. The colour intensity indicates the degree of protein presence, where high relative abundance is indicated in red and low relative abundance in blue. The rows in the heat map show the detected proteins in four different growth conditions. The columns show the electron donors used by *G. sulfurreducens* organized from left to right as formate, acetate, hydrogen and mix (containing all three electron donors). Protein abbreviations are: type II secretion system pseudopilin oxpG (OxpG), pilin domain 1 protein pilA (PliA-N), pilin domain 2 protein (PilA-C), thiamin biosynthesis protein ThiI-related adenine nucleotide alpha hydrolase superfamily protein (GSU0434), type IV pilus biogenesis ATPase PilB(PilB), type IV pilus inner membrane protein PilC (pilC), type IV pilus biogenesis ATPase PilM (PilM), type IV pilus biogenesis protein PilN (PilN), type IV pilus biogenesis protein PilO (PilO), type IV pilus secretin lipoprotein PilQ (PilQ), type IV pilus assembly lipoprotein PilP (PilP), sensor histidine kinase PilS (PAS, HisKA, HATPase_c) (PilS), sigma-54-dependent transcriptional response regulator PilR (REC, sigma54 interaction, HTH8) (PilR), twitching motility pilus retraction protein (pilT-1), twitching motility pilus retraction protein (PilT-4).

**Table S1.** Cultivation conditions for *G. sulfurreducens* used in this study. The growth conditions shown in bold were used for proteomic analysis.

| Growth conditions | Acetate  (mM) | Formate  (mM) | Hydrogen  (mM) | Fe(III) citrate  (mM) |
| --- | --- | --- | --- | --- |
| **Acetate** | 5 | - | - | 50 |
| **Formate** | - | 20 | - | 45 |
| **Hydrogen** | - | - | 10 mM | 25 |
| Acetate + formate | 5 | 20 | - | 80 |
| Hydrogen + acetate | 5 | - | 10 mM | 60 |
| Hydrogen + formate |  | 20 | 10 mM | 60 |
| **Hydrogen + acetate+ formate** | 5 | 20 | 10 mM | 80 |
